# Investigation of Ultrasound-Mediated Intracellular Ca^2+^ Oscillations in HIT-T15 Pancreatic β-Cell Line

**DOI:** 10.3390/cells9051129

**Published:** 2020-05-04

**Authors:** Chi Woo Yoon, Nan Sook Lee, Kweon Mo Koo, Sunho Moon, Kyosuk Goo, Hayong Jung, Changhan Yoon, Hae Gyun Lim, K. Kirk Shung

**Affiliations:** 1 Department of Biomedical Engineering, University of Southern California, Los Angeles, CA 90089, USA; soulishguy@gmail.com (C.W.Y.); nanlee@usc.edu (N.S.L.); kweonmok@usc.edu (K.M.K.); sunhomoo@usc.edu (S.M.); gooky01@gmail.com (K.G.); hayong@usc.edu (H.J.); yoonch80@gmail.com (C.Y.); kkshung@usc.edu (K.K.S.); 2 Department of Biomedical Engineering, Inje University, Gimhae, Gyeongnam 50834, Korea; 3 Department of Creative IT Engineering and Future IT Innovation Lab, Pohang University of Science and Technology, Pohang, Gyeongbuk 37673, Korea

**Keywords:** ultrasound, calcium oscillations, pancreatic β-cells, purinergic signaling, hemichannels, ATP

## Abstract

In glucose-stimulated insulin secretion (GSIS) of pancreatic β-cells, the rise of free cytosolic Ca^2+^ concentration through voltage-gated calcium channels (VGCCs) triggers the exocytosis of insulin-containing granules. Recently, mechanically induced insulin secretion pathways were also reported, which utilize free cytosolic Ca^2+^ ions as a direct regulator of exocytosis. In this study, we aimed to investigate intracellular Ca^2+^ responses on the HIT-T15 pancreatic β-cell line upon low-intensity pulsed ultrasound (LIPUS) stimulation and found that ultrasound induces two distinct types of intracellular Ca^2+^ oscillation, fast-irregular and slow-periodic, from otherwise resting cells. Both Ca^2+^ patterns depend on the purinergic signaling activated by the rise of extracellular ATP or ADP concentration upon ultrasound stimulation, which facilitates the release through mechanosensitive hemichannels on the plasma membrane. Further study demonstrated that two subtypes of purinergic receptors, P_2_X and P_2_Y, are working in a competitive manner depending on the level of glucose in the cell media. The findings can serve as an essential groundwork providing an underlying mechanism for the development of a new therapeutic approach for diabetic conditions with further validation.

## 1. Introduction

Insulin, a peptide hormone secreted by the pancreas, plays a crucial role in maintaining the homeostasis of the blood glucose levels in the human body. It lowers the blood glucose levels by facilitating glucose uptakes in the liver, skeletal muscle, and adipose tissue. Failure to maintain blood glucose levels, also known as hyperglycemia, can give rise to many different severe health conditions, including diabetes. Insulin secretion is stimulated by many different hormones and neurotransmitters such as glucagon-like peptide-1 [1] and carbon monoxide [2], but glucose is a major secretagogue.

Over the years, the glucose-stimulated insulin secretion (GSIS) has been extensively studied, and its mechanism is well documented [3,4]. An increase in blood glucose concentration accelerates metabolism in pancreatic β-cells, leading to an elevated cytoplasmic ATP/ADP ratio. ATP-sensitive K^+^ channels respond to the elevated ATP/ADP ratio in the cytoplasm by closing their channels, resulting in the depolarization of the cell’s membrane potential. This depolarization opens voltage-dependent Ca^2+^ channels (VDCCS), allowing a transient influx of Ca^2+^ from extracellular space. In turn, the elevated free cytosolic Ca^2+^ concentration triggers the release of insulin granules.

Furthermore, it has been known that the insulin secretion occurs in a pulsatile manner [5], and the oscillatory release of insulin helps to maintain insulin sensitivity in target cells. Without the oscillatory insulin secretion pattern, more insulin is required to achieve the same effect [6,7,8], possibly causing insulin resistance from the recipient cells. In fact, it was reported that insulin oscillations were diminished from patients with type 1 [9] and type 2 diabetes [10]. Interestingly, the oscillatory insulin secretion is not only found in the pancreas but also from isolated individual pancreatic islets, and even from single β-cells [11]. In a single β-cell, oscillations of the cytosolic Ca^2+^ concentration, which mainly originated from the glucose metabolism, synchronize with the pulsatile insulin secretion. 

There have been numerous reports suggesting that GSIS does not rely solely on the K_ATP_ channel-dependent mechanism, and mechanosensitive mechanisms may also be involved. There are a few hypotheses on mechanosensitive pathways, such as volume-regulated anion channels [12] or mechanosensitive transient receptor potential (TRP) channels [13]. However, the level of intracellular Ca^2+^ plays a fundamental role in all suggested pathways. Recently, Castellanos et al. [14] reported the Ca^2+^-dependent insulin release from rat INS 832/13 β-cells upon ultrasound stimulation, suggesting a possible therapeutic intervention of the diabetic condition using mechanical energy-based modality. 

For the last few years, our group has reported that focused ultrasound can induce intracellular Ca^2+^ elevations in cancer cell lines [15,16], human umbilical vein endothelial cells (HUVECs) [17], and human mesenchymal stem cells (hMSCs) [18]. In the recent report, we suggested the concept of an ultrasound-based intracellular Ca^2+^ signaling modulator, which possesses the capability to regulate downstream Ca^2+^-dependent cellular processes non-invasively and remotely. In the present study, we explored the ultrasound-evoked intracellular Ca^2+^ dynamics of single cells from the clusters of a clonal HIT-T15 pancreatic β-cell line using a 45-MHz focused ultrasound. The results indicate that ultrasound can induce intracellular Ca^2+^ oscillations via purinergic signaling. Two distinctive oscillatory Ca^2+^ patterns, fast-irregular and slow-periodic, were found in the cells stimulated by low-intensity ultrasound, and they have shown a competitive manner depending on the level of glucose. Further study revealed that the fast-irregular mode of Ca^2+^ oscillation depends on both P_2_X receptors and L-type voltage-dependent Ca^2+^ channels, while the slow-periodic mode relies on P_2_Y receptors and store-operated Ca^2+^ channels.

## 2. Materials and Methods

### 2.1. Reagents and Inhibitors

Ham’s F12K special (with 1.5 g/L sodium bicarbonate) was obtained from the USC Norris Comprehensive Cancer Center Core (Los Angeles, CA, USA). Dialyzed donor equine serum was obtained from Rocky Mountain Biologicals (Missoula, MT, USA). Heat-inactivated fetal bovine serum (HI-FBS), Penicillin–Streptomycin, l-glutamine, Fluo-4 AM, and Calcein AM were purchased from Thermo Fisher Scientific (Cambridge, MA). Apyrase, carbenoxolone (CBX), cyclopiazonic acid (CPA), lanthanum (La^3+^), nifedipine, Pluronic F-127, pyridoxalphosphate-6-azophenyl-2′,4′-disulfonic acid (PPADS), and suramin were purchased from Sigma-Aldrich (St Louise, MO, USA).

### 2.2. Cell Preparation

The HIT-T15 cell line was purchased from ATCC (Manassas, VA, USA) and maintained in the complete growth medium with following ingredients: Ham’s F12K with 2 mM l-glutamine, 10% dialyzed donor equine serum, 2.5% HI-FBS, and 1% Penicillin–Streptomycin. The HIT-T15 cells were seeded at a density of 1.3 × 10^5^ cells/cm^2^ and cultured in 5% CO_2_ at 37 °C. The flesh complete media was replenished every 2–3 days, and the cells were passaged every week. For this study, only the cells with passage numbers between 20 and 30 were used.

### 2.3. Ultrasound Stimulation

For this project, a press-focused 45-MHz single element lithium niobate (LiNbO_3_) transducer was designed and fabricated in house following a protocol previously described [19]. Among various piezoelectric materials, LiNbO_3_ was selected because it holds many advantages such as good electromechanical coupling, a low dielectric constant, and high longitudinal sound speed [20]. In the design, the size of a cluster of HIT-T15 cells was primarily considered to generate a beamwidth comparable to a single cluster (10–20 cells), and it was achieved by the press-focusing technique (f-number: 2.2). A calibrated capsule type hydrophone with 20 dB preamplifier (HGL-0085/AH-2010, Onda Corp., Sunnyvale, CA) was employed to assess the actual performance of the fabricated transducer. A lateral beam-width (115 μm) was estimated by measuring the distance between the 6 dB points below the maximum. Note that a theoretical lateral resolution for the transducer was 75 μm ($R_{L}=f\#\times \lambda ),$ and the measured value might be overestimated since the hydrophone’s electrode aperture size (85 μm) was not optimal to measure high-frequency transducers (>40 MHz) accurately. In this study, the transducer was driven by a fixed 18 V peak-to-peak voltage, pulse repetition frequency at 1 kHz, and duty cycle at 2% (I_SPTA_: 113.1 mW/cm^2^) to be in the realm of low-intensity pulsed ultrasound. The cytotoxicity of ultrasound stimulation was examined using a viability dye, calcein AM. No indication of compromised viability was observed up to 60 h after the ultrasound exposure (Supplementary Figure S1).

### 2.4. Live Intracellular Ca^2+^ Imaging

The clonal HIT-T15 cells were seeded on 35 mm culture dishes at a density of 2 × 10^5^ cells/cm^2^ and kept in the CO_2_ incubator for 48 h before each experiment. For the imaging solutions, mainly modified Hank’s balanced salt solution with Ca^2+^ and Mg^2+^ (HBSS+) containing 11.1 mM D(+) glucose was used, but HBSS+ containing 2.8 mM and 5.5 mM D(+) glucose were also used as needed. The HIT-T15 cells on 35 mm culture dish were washed with HBSS+ once and incubated with 2 μM of Fluo-4 AM in room temperature for 30 min for Ca^2+^ imaging. After the incubation, the dish was washed three times and imaged with an epi-fluorescence inverted microscope (IX71, Olympus America Inc., Center Valley, PA, USA). Fluorescence images were acquired either for 30 min at 0.5 frames per second or for 5 min at 1 frame per second.

### 2.5. Data Processing and Statistics

Acquired stacked images were processed with CellProfiler image analysis software [21] using a customized pipeline to locate single cells and collect fluorescence intensities automatically. The extracted intensities were loaded in Matlab (Mathworks) for normalization (ΔF/F) and for counting the number of cells showing active Ca^2+^ dynamics (defined as cells with ΔF/F_max_ greater than basal noise level by 2-fold) with and without ultrasound exposure. The percentage of responding cells was calculated by the active cells divided by the total number of cells in each image field. In addition, the period of Ca^2+^ oscillations was measured and compared in the cells, either bathing in 5.5 mM glucose or inhibitors that suppressed the fast-irregular oscillations. Due to the nature of irregular oscillations, the period of oscillations cannot be measured in the fast oscillations.

## 3. Results

### 3.1. Intracellular Ca^2+^ Dynamics in HIT-T15 Cells upon Various Stimuli

We first investigated intracellular Ca^2+^ dynamics in HIT-T15 cells using a high K^+^ (40 mM) extracellular buffer. The high K^+^ stimulation has been used to depolarize the cell membrane in order to activate VDCCs on the membrane and allow an influx of Ca^2+^. A sudden increase of intracellular Ca^2+^ was observed as soon as the imaging solution was replaced by the high K^+^ buffer (Supplementary Figure S2a). The result indicates that the VDCCs on the membrane were activated by the altered K^+^ concentration gradient between the inside and outside of the cells and allow an influx of Ca^2+^ from the outside. Furthermore, the gradual decrease indicates that the cells’ machinery Ca^2+^ pumps are functioning.

Next, the HIT-T15 cells were stimulated with a high concentration of glucose to monitor the glucose-induced Ca^2+^ activity. The cells were maintained in HBSS+, and at t = 600 s, it was replaced with high glucose (17 mM) buffer solution. The cells responded to the high glucose with oscillatory Ca^2+^ signaling (Supplementary Figure S2b). The oscillations in intracellular Ca^2+^ are known to synchronize with the oscillatory metabolism of the β-cell and in turn create pulsatile secretion of insulin [22]. The pulsatile insulin secretion gives a means of lowering total insulin amount to maintain the blood glucose level compared to a constant rate of secretion [7]. 

**Figure 1:**
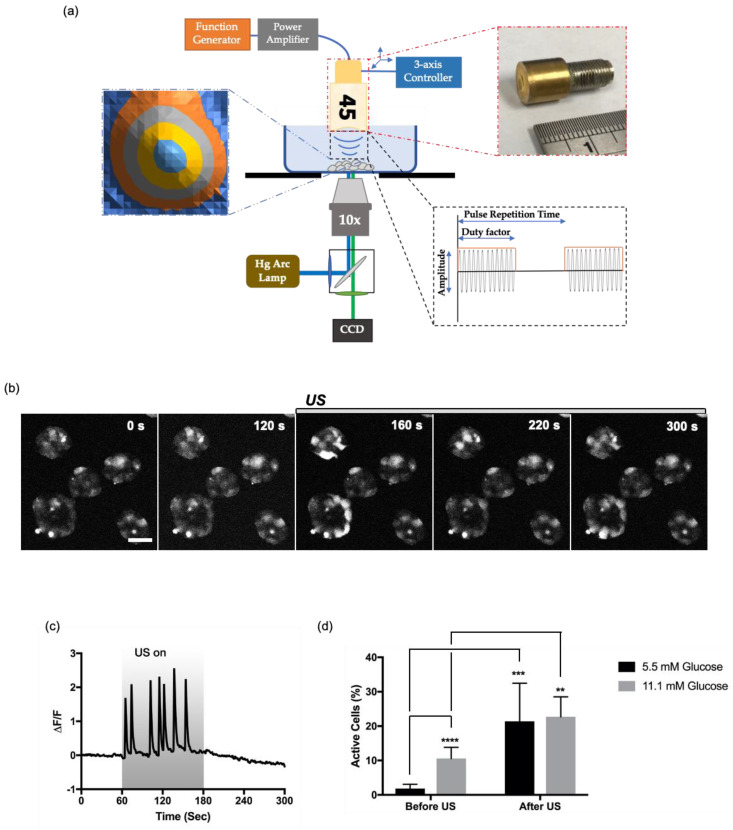
Ultrasound-induced intracellular Ca^2+^ dynamics in HIT-T15 pancreatic β-cells. (**a**) Schematic diagram of an ultrasound stimulation system. A pulsed sine wave was generated by a function generator and amplified by a power amplifier to drive a 45-MHz focused transducer. The position of the transducer was controlled by a three-axis stage controller. The focal distance from the transducer to the surface of culture dish was aligned using a pulser–receiver. Ultrasound-induced intracellular Ca^2+^ dynamics were monitored by an inverted epifluorescence microscope. (**b**) Time lapse images of HIT-T15 cells before and after ultrasound stimulation. Scale bar 50 µm. (**c**) Ultrasound-induced Ca^2+^ oscillation from a single HIT-T15 cell. The cells were bathed in HBSS+ with 11.1 mM glucose. The gray box indicates the duration of ultrasound exposure. (**d**) Percentage of cells active in Ca^2+^ signaling before and after ultrasound stimulation. The cells were adapted to different glucose concentrations, 5.5 mM and 11.1 mM, for 1 h before the imaging. The error bar indicates S.D. **, ***, and **** indicate *p*-value less than 0.01, 0.001, and 0.0001, respectively.

To test whether ultrasound stimulation can also evoke intracellular Ca^2+^ oscillations from resting cells as shown in the high-glucose stimulation, a cluster of HIT-T15 cells was exposed to 45-MHz pulsed ultrasound. In this study, the power (I_SPTA_) of the ultrasound was fixed at 113.1 mW/cm^2^ (input voltage: 60 mV, pulse repetition time: 1 ms, duty factor: 2%) to be in the range of low-intensity and also comparable to our previous reports [16,18]. The ultrasound stimulation setup is illustrated in Figure 1a. 

Ultrasound induced fast Ca^2+^ oscillations in the HIT-T15 cells (Figure 1c). The periodicity of oscillations was irregular, with the timescale of each Ca^2+^ spike less than 20 s. The ultrasound-induced Ca^2+^ dynamics are comparable to fast-irregular oscillations observed in a stepwise increase in glucose concentration that mimics the transition from fasting to feeding states [23]. 

Although the ultrasound beam was focused on a targeted cluster of cells, only a fraction of cells in the cluster responded. In general, cells on the edges of clusters responded well, while cells on the center did not (Figure 1b). Moreover, ultrasound-induced Ca^2+^ responses were monitored from non-targeted cells (cells located outside of ultrasound beam) throughout all over the image field, suggesting the involvement of intercellular signaling (Supplementary Video S1). To quantify the number of cells responded to ultrasound, fluorescence imaging was performed for 300 s with the HIT-T15 cells exposed to ultrasound between t = 150 s and t = 300 s. Then, the numbers of cells showing Ca^2+^ dynamics before and after ultrasound stimulation were compared as percentages. As illustrated in Figure 1d, the percent of cells showing Ca^2+^ dynamics (defined by active cells) was increased by 10-fold, 1.85 ± 1.22% to 21.40 ± 11.08% (*n* = 11, *p* = 0.0002), in the cells bathed with normal HBSS+ containing 5.5 mM glucose. However, when the cells were bathed in HBSS+ containing 11.1 mM glucose (stimulating concentration), the percent of active cells before ultrasound was 10.60 ± 3.21%, and the number was increased by 2-fold with the ultrasound stimulus to 22.72 ± 5.78% (*n* = 6, *p* = 0.0025, Figure 1d). Still, the percentages of the active cells were low, which could be due to (1) the heterogeneity of the HIT-T15 cell line [24] and (2) the quantification method, which excluded cells showing Ca^2+^ dynamics in low amplitude (less than 2-folds from the basal level).

Overall, the results indicate that ultrasound can activate Ca^2+^ responses from otherwise resting cells both in the basal and stimulating level of glucose.

### 3.2. Two Distinctive Ultrasound-Induced Ca^2+^ Oscillations

**Figure 2:**
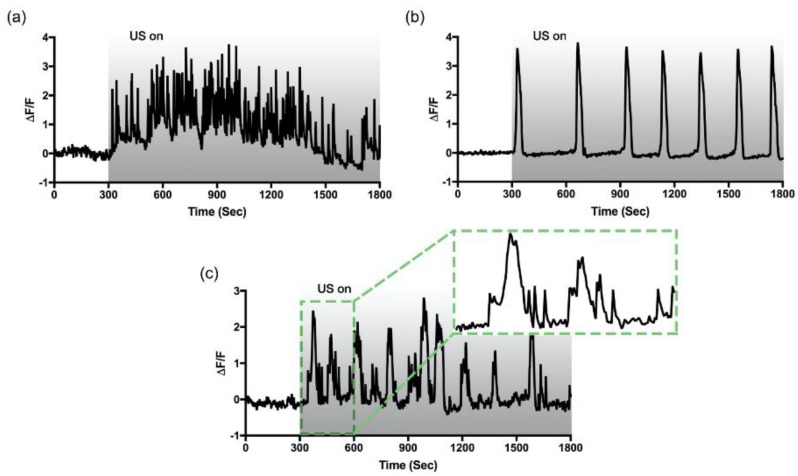
Different patterns of intracellular Ca^2+^ oscillations evoked by ultrasound. Clusters of HIT-T15 cells were exposed to low-intensity pulsed ultrasound for 25 min starting at t = 300 s. The gray bar indicates the duration of ultrasound exposure. Fast-irregular (**a**), and slow-periodic (**b**) oscillation patterns were dominantly observed from the cells adapted to 11.1 mM (stimulating) and 5.5 mM (non-stimulating) of glucose, respectively. Seldomly, mixed (**c**) oscillation patterns were also monitored in the both conditions.

HIT-T15 cells showed the majority of the fast-irregular oscillations in a bath of 11.1 mM glucose (Figure 2a). However, when the HIT-T15 cells were adapted to 5.5 mM glucose, the majority of the fast-irregular oscillations was replaced by single Ca^2+^ spikes with a longer timescale of 30–50 s. To monitor the Ca^2+^ dynamic for an extended period of time, we increased the imaging duration to 30 min. Surprisingly, another pattern of oscillatory Ca^2+^ dynamics was found, which has a slow but periodic oscillatory pattern (period: 231 ± 92 s), as shown in Figure 2b. Leech et al. described the appearance of the slow Ca^2+^ oscillation in a steady-state concentration of glucose [23]. Seldomly, a third type of oscillation was also found, which can be described as fast oscillations superimposed on the slow oscillation (Figure 2c); this is also known as the mixed type [25]. Aside from the frequency and periodicity differences, it was observed that the baseline for the fast-irregular oscillation fluctuates, while the baseline for the slow-periodic oscillation remains relatively constant. These data suggested that multiple pathways may be involved in ultrasound-induced Ca^2+^ oscillations, and the appearance of each oscillatory pattern depends on the level of glucose to which the HIT-T15 cells were adapted.

### 3.3. The Involvement of Purinergic P_2_ Signaling

Purinergic signaling plays a regulatory role in the endocrine system. Many different subtypes of purinergic receptors are expressed in secretory cells to meditate hormone release [26]. In the pancreatic β-cells, ATP is released either from nerve terminals [27,28] or secretory granules [29], and it plays several physiological roles [30]. We hypothesized that ultrasound induces the oscillatory Ca^2+^ responses by facilitating ATP release via mechanosensitive hemichannels based on our previous study [18]. 

**Figure 3:**
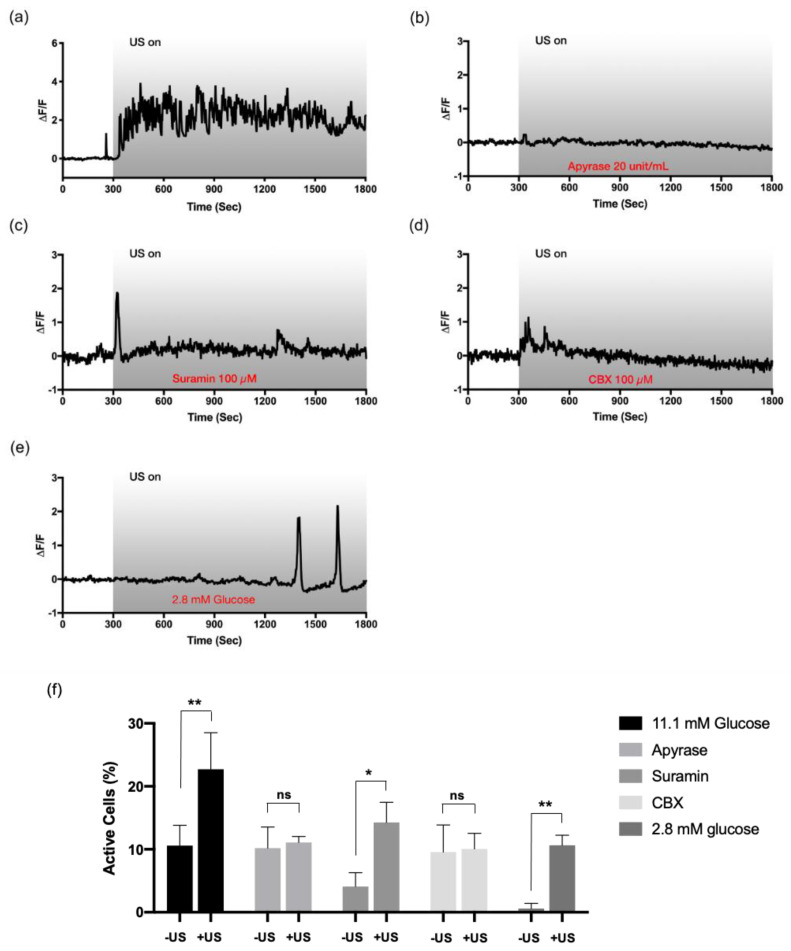
Involvement of purinergic signaling and hemichannels in the ultrasound-evoked Ca^2+^ mobilization. The HIT-T15 cells pre-incubated (**a**) without inhibitor (control) or with (**b**) apyrase (degrades extracellular ATP, 20 unit/mL), (**c**) suramin (P_2_ receptor inhibitor, 100 µM), and (**d**) carbenoxolone (CBX) (hemichannel inhibitor, 100 µM) were stimulated by ultrasound to investigate the underlying pathway. In all cases, ultrasound-induced Ca^2+^ dynamics were abolished, indicating the involvement of purinergic receptors triggered by ATP released from hemichannels. All experiments were carried out with modified HBSS+ containing 11.1 mM glucose except (**e**) where HBSS+ with 2.8 mM glucose was used. Delayed Ca^2+^ oscillations were monitored when the cells were adapted to low glucose. (**f**) Percentage of cells showing Ca^2+^ dynamics before and after ultrasound stimulation. Only groups pretreated with suramin and 2.8 mM glucose showed statistically significant increases upon ultrasound, yet they were either single peaks (suramin) or delayed oscillations (low glucose). * and ** indicate *p*-values less than 0.05 and 0.01, respectively. *n* = 3 for all groups.

To verify the hypothesis, the HIT-T15 cells were incubated with apyrase (20 units/mL, 10 min), which is known to degrade extracellular ATP. Bathing with apyrase significantly abolished the ultrasound-induced Ca^2+^ dynamic (Figure 3b,f). A non-selective P_2_ receptor antagonist, suramin (100 μM, 10 min), also blocked the ultrasound-induced oscillations, suggesting the involvement of P_2_ receptors in the pathway (Figure 3c,f). Interestingly, the basal Ca^2+^ activity was also greatly suppressed to 4.09 ± 2.20% with suramin (approximately 10% without the drug) and a single peak appeared upon ultrasound stimulation. As for the source of extracellular ATP, mechanosensitive hemichannels were found to be responsible for the inhibitory effect of CBX (100 μM, 30 min, Figure 3d,f). The overall pathway of ultrasound-induced Ca^2+^ responses in the HIT-T15 cells was consistent with the findings from the hMSC model [18]. It is worth noting that with the inhibitors, both ultrasound-induced fast-irregular and slow-periodic oscillations disappeared from the cells adapted to 11.1 mM glucose.

If both modes of the ultrasound-induced Ca^2+^ oscillations depend on ATP release, the next question was: how do the cells choose one pattern over another? We focused on the fact that two subtypes of purinergic receptors, ionotropic (P_2_X) and metabotropic (P_2_Y), have different sensitivity to a range of ATP or ADP concentrations [31]. Since the amount of intracellular ATP would largely determine the amount of ATP release, we bathed the cells in HBSS+ containing 2.8 mM glucose for adaptation and stimulated with ultrasound. Interestingly, the cells adapted to the low glucose lost spontaneous Ca^2+^ activities before ultrasound exposure, and delayed Ca^2+^ responses were observed upon ultrasound stimulation; yet, once it appeared, the slow-periodic oscillatory pattern persisted (Figure 3e). The result is consistent with our hypothesis that the level of glucose available in the media, which, in turn, could affect the amount of intracellular ATP in the cells, works as a modulatory signal to determine the patterns.

### 3.4. Fast and Irregular Oscillation Depends on P_2_X Receptors Coupled to L-Type Ca^2+^ Channels

**Figure 4:**
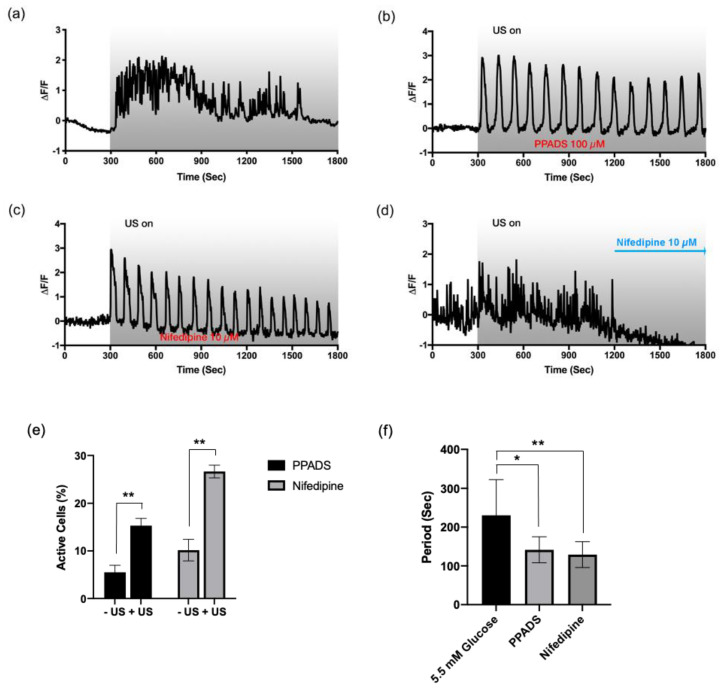
Fast and irregular Ca^2+^ oscillations depend on L-type voltage-dependent Ca^2+^ channels coupled to P_2_X purinergic receptors. Ultrasound-evoked Ca^2+^ dynamic profiles from the HIT-T15 cells (**a**) without drug (control), or in the presence of (**b**) pyridoxalphosphate-6-azophenyl-2′,4′-disulfonic acid (PPADS) (100 μM, pretreated), (**c**) nifedipine (10 μM, pre-treated), and (**d**) nifedipine (10 μM, added at 1200 s). Fast and irregular oscillation patterns disappeared with PPADS (**b**) and nifedipine (**c**,**d**); instead, slow-periodic patterns appeared. All experiments were carried out with the modified HBSS+ containing 11.1 mM glucose. (**e**) Percentage of active cells before and after ultrasound stimulation. *n* = 3. (**f**) Oscillation frequency analysis. In comparison to the oscillations found from the cells adapted to 5.5 mM glucose, the oscillations found from the PPADS and nifedipine bathed groups. *n* = 10. * and ** indicate *p*-values less than 0.05 and 0.01, respectively.

To investigate the subtype of purinergic receptors responsible for the fast-irregular oscillation, we first incubated the HIT-T15 cells with HBSS+ containing 11.1 mM glucose in the presence of PPADS (100 µM, 30 min), which is a selective purinergic P_2_X receptor blocker at the tested concentration. Interestingly, the fast-irregular oscillations pattern shown in 11.1 mM glucose (Figure 4a) was converted to the slow-periodic oscillatory pattern (period: 142 ± 34 s) under the inhibition of purinergic P_2_X receptors (Figure 4b,f). This result suggests the correlation of the P_2_X receptors with the fast-irregular oscillation pattern.

Although the P_2_X receptors work as an ion channel allowing the influx of Ca^2+^ and Na^2+^, the rapid oscillatory pattern cannot be explained solely by the action of P_2_X. Previous studies often suggested the role of L-type VDCC in glucose-induced insulin secretion [23,32]. To assess the role of L-type VDCC in the fast Ca^2+^ oscillation, nifedipine (10 μM) was added to the HBSS+ containing 11.1 mM glucose. When the HIT-T15 cells were pre-bathed with nifedipine, only the periodic Ca^2+^ oscillations (period: 129 ± 33 s) were observed (Figure 4c,f), similar to with the P_2_X inhibitor. To confirm, nifedipine was added while imaging, and the fast-irregular Ca^2+^ oscillation quickly disappeared (Figure 4d). This result strongly suggests the involvement of L-type VDCC in the fast-irregular Ca^2+^ oscillation induced by ultrasound. It is worth noting that the periods of oscillations found from the cell bathed with both PPADS and nifedipine were smaller (oscillate faster) than the slow-periodic oscillations observed from 5.5 mM glucose groups (Figure 4f). This could be due to the higher ATP level available from the cells bathed with 11.1 mM glucose.

### 3.5. Slow and Periodic Oscillation Depends on P_2_Y Receptors and Store-Operated Ca^2+^ Channels

The expression of both subtypes of purinergic receptors, P_2_X and P_2_Y, was reported previously in pancreatic β-cells [33], including HIT-T15 cells [34]. The P_2_Y receptors are metabotropic receptors coupled to the G-protein, which produces secondary messenger IP_3_ from PIP_2_ and in turn initiates the release of Ca^2+^ via the IP_3_ receptor. Based on the fact that the presence of apyrase and suramin inhibited both patterns of Ca^2+^ oscillation (Figure 3b,c), but not with PPADS (Figure 4b), we concluded that the slow-periodic Ca^2+^ oscillation relies on P_2_Y receptors.

**Figure 5:**
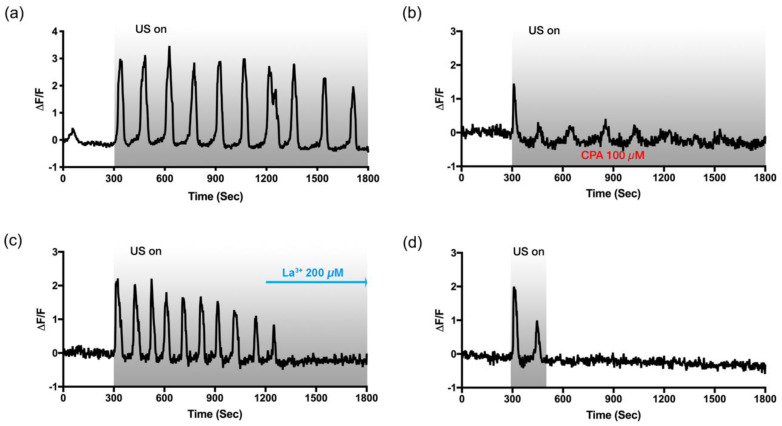
Slow and periodic Ca^2+^ oscillations depend on the store-operated Ca^2+^ entry pathway. Intracellular Ca^2+^ dynamics in response to ultrasound exposure between t = 300 s and t = 1800 s (**a**) without drug (control), or bathing with (**b**) CPA (100 µM, pre-treated), (**c**) La^3+^ (200 µM, added at 1200 s). (**d**) To verify the primary role of P_2_Y for the Ca^2+^ release, cells were stimulated for 3 min (300 s–480 s). All experiments were carried out with the regular HBSS+ containing 5.5 mM glucose.

As to confirm the role of P_2_Y in the ultrasound-induced slow Ca^2+^ oscillation, the HIT-T15 cells, adapted to 5.5 mM glucose, were treated with CPA (100 μM, 30 min), a sarco/endoplasmic reticulum Ca^2+^ ATPase (SERCA) inhibitor, to deplete Ca^2+^ storage. As shown in Figure 5b, the Ca^2+^ response was greatly diminished after the first Ca^2+^ peak, suggesting that the slow-periodic Ca^2+^ oscillation depends on Ca^2+^ release from the storage, and without the functioning Ca^2+^ pump for replenishing the storage, Ca^2+^ oscillation cannot be sustained. 

The store-operated Ca^2+^ channels (SOCs) on the plasma membrane often serve as a Ca^2+^ replenishing mechanism. When Ca^2+^ is released from intracellular storage, such as the endoplasmic reticulum (ER), SOCs are activated and allow Ca^2+^ entry into the cytoplasm and the storage. SOCs also play a critical role in GSIS [35]. La^3+^, a trivalent cation that inhibits the SOCs, was added while monitoring the ultrasound-induced Ca^2+^ oscillations to investigate the relevance of SOCs in this pathway. As the SOCs were inhibited by La^3+^ (200 μM, added at t = 1200 s), the oscillatory Ca^2+^ response disappeared, as depicted in Figure 5c. Lastly, we stimulated the cells only for 3 min (instead of 25 min) to see if the continuous exposure of ultrasound would be necessary for the sustained oscillations. It was found that the oscillation patterns disappeared as soon as the ultrasound stimulation was stopped (Figure 5d). This indicates that continuous exposure to ultrasound is required to release Ca^2+^ from the storage, but SOC itself cannot sustain the oscillatory Ca^2+^ mobilization.

## 4. Discussion

In the present study, we investigated the cellular responses of the pancreatic HIT-T15 β-cells to 45-MHz low-intensity focused ultrasound by monitoring the ultrasound-induced intracellular Ca^2+^ dynamics in live cells. The HIT-T15 cell line was initially produced by the SV40 transformation of hamster pancreatic islet cells and has been widely used as the β-cell study model, since it secretes insulin in response to a variety of insulin secretagogues such as glucose, glucagon, and methylxanthine [36]. Upon exposure to ultrasound, it was found that the resting HIT-T15 cells were activated and displayed the oscillatory Ca^2+^ responses via purinergic signaling, and a mechanosensitive hemichannel was found to be responsible for releasing ATP into extracellular space.

We are not the first to investigate how β-cells respond to ultrasound stimulation. Castellanos et al. [14,37] reported insulin releases from INS-1 832/13 rat insulinoma cell line in response to 800 kHz of ultrasound stimulation (continuous ultrasound, I_SATA_ = 1 W/cm^2^). In another sequential paper, the authors monitored ultrasound-induced secretary events by carbon fiber amperometry and detected sustained amperometric peaks, which prolong the duration of ultrasound stimulation [14]. In addition, they showed that the ultrasound-induced secretary events are depending on the influx of Ca^2+^ by showing that chelating extracellular Ca^2+^ from the imaging buffer significantly reduced the peaks. However, despite the similarity in the concept, the overall experimental design and stimulation parameters used in our study were mostly different. First, the acoustic output utilized in the present study was considerably lower (pulsed ultrasound, I_SPTA_ = 113.1 mW/cm^2^), which may have resulted in an entirely different Ca^2+^ mobilizing pathway. More importantly, we aimed attention at spatiotemporal Ca^2+^ responses using a high-frequency focused ultrasound at 45 MHz, which offered a way to distinguish between directly stimulated cells from their adjacent cells and also allowed us to obtain a better understanding of the underlying mechanism in at the single-cell level. Previously, we have shown that both 3-MHz and 38-MHz ultrasound stimulation can induce Ca^2+^ responses in cancer cells, indicating that the mechanotransduction pathways reported in this study may not be limited to high-frequency ultrasound [16].

The biological effects of ultrasound can be largely divided into two categories: thermal bioeffects and mechanical bioeffects. However, we excluded the possibility of thermal effects because the intensity level was too low (I_SPTA_ = 113.1 mW/cm^2^), and acoustic absorption in the cell layer should be minimal, as the absorption correlates with the distance acoustic wave travels [38,39]. Therefore, we believe that the mechanical perturbation created by acoustic radiation force is the dominant factor in our experimental setting. Additionally, the formation of a standing wave could be another possible source of the mechanical perturbation [40]. However, in theory, an adjacent node will be formed 17 µm (λ/2) above the culture dish and may not affect directly, as the height of adherent cells are generally considered to be shorter [41,42]. The oscillatory pattern of intracellular Ca^2+^ has been extensively studied in pancreatic β-cells, since its dynamic is tightly coupled to the exocytosis of insulin-containing vesicles from single cells, and its physiological significances have been documented [43]. Several modes of Ca^2+^ oscillation have been reported in β-cells through different pathways of Ca^2+^ entry depending on the concentration of glucose or the presence of other stimuli [44]. To the best of our knowledge, we are the first to report ultrasound-induced intracellular Ca^2+^ oscillations in the pancreatic β-cells. It was found that exposure to ultrasound induces Ca^2+^ dynamics even from the cells that did not respond to the stimulating level of glucose. Note that regardless of the glucose levels to which cells were adapted, the percentages of active cells were consistent after the stimulation (approximately 22%, Figure 1d), suggesting that ultrasound-induced Ca^2+^ responses may not share the same pathway with glucose-stimulated Ca^2+^ responses.

Interestingly, two very distinct modes of oscillations were dominantly observed where the cells adapted to different levels of glucose (5.6 mM and 11.1 mM). Further investigation with pharmaceutical agents revealed that the two oscillatory Ca^2+^ responses were originated from the activations of the two subtypes of P_2_ purinergic receptors. While P_2_X receptors are known for responding to only ATP and its derivatives, a few P_2_Y receptors, namely P_2_Y_1_, P_2_Y_12_, and P_2_Y_13_, have generally higher affinity to ADP and its derivatives than ATP [45,46]. Considering β-cell’s intrinsic property that tightly regulates ATP production in response to glucose level fluctuation [47], we believe that the amount of ATP (or ATP/ADP ratio) in cytoplasm plays a pivotal role selecting one pathway over another.

**Figure 6:**
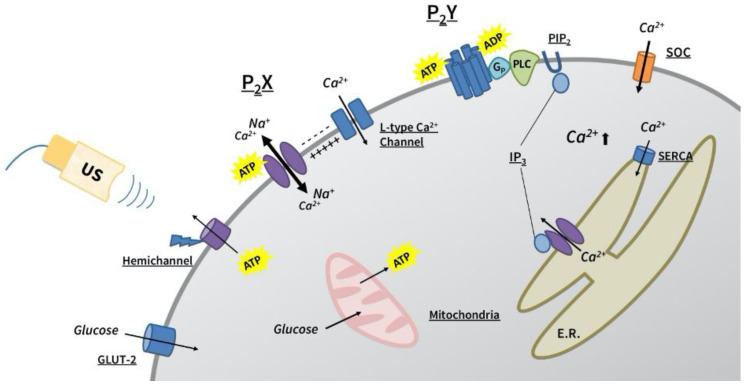
A schematic drawing representing the proposed pathways of ultrasound-evoked Ca^2+^ dynamics in the HIT-T15 cells. Mechanosensitive hemichannel releases ATP and/or ADP into extracellular space in response to ultrasound stimulation, which in turn activates both P_2_ receptors. Depending on the amount of released ATP or ADP, the two subtypes of P_2_ receptors, P_2_X and P_2_Y, will be activated in a competitive manner, evoking either fast-irregular or slow-periodic Ca^2+^ oscillations, respectively.

The fast-irregular oscillations found from this study showed very distinctive Ca^2+^ profiles, an instant rise followed by a relatively slow fall and fluctuations of the baseline, which can be often found from Ca^2+^ mobilization via VDCCs (Figure 2a). The spiking pattern was abolished or replaced by the slow-periodic oscillations in the presence of P_2_X inhibitor PPADS and L-type Ca^2+^ channel blocker, nifedipine, indicating that both channels are required for the specific pattern of Ca^2+^ entry. Although direct evidence is not presented in this paper, it seems that membrane depolarization is caused by ion exchange through the P_2_X channels, which in turn activates the VDCC. In fact, the P_2_X receptor-mediated depolarization was found to be sufficient to open up the VDCC in human pancreatic β-cells [48]. The overall suggested mechanisms of ultrasound-induced Ca^2+^ oscillations are illustrated in Figure 6.

The finding that L-type Ca^2+^ channels are closely linked to the fast-irregular oscillation pattern gives us another insight into the competing aspect of P_2_X and P_2_Y activations observed from this study. Gong et al. [49] reported the inhibitory effect of extracellular ATP on L-type Ca^2+^ channel currents through the P_2_Y-dependent pathway in rat pancreatic β-cells. A similar line of evidence was also found from myocytes [50,51], and the inhibition was reversed in the presence of protein kinase C (PKC) antagonist, indicating that PKC activation via G-protein-coupled P_2_Y signaling cascade is the source of the inhibition. It was found that activated PKC phosphorylates two sites on the *n* terminal of the L-type Ca^2+^ channel, which leads to the blockage of the channels by changing a net charge [52]. 

The mechanosensitive characteristic of the non-junctional hemichannels was reported elsewhere [53,54,55], and a recent study proposed that the association with integrin is critical for the sensing [56]. In fact, several lines of evidence support the finding that ATP can be released via mechanosensitive hemichannels. We have previously shown that the connexin 43 hemichannel works as a mechanosensor in hMSC that releases ATP to the extracellular space in response to low-intensity ultrasound [18]. In addition, we recently showed that ultrasound-induced ATP release was reduced significantly when the pannexin 1 hemichannel was either inhibited or knocked down in the prostate PC-3 cancer cells [57]. Ultrasound is a very unique modality that can transmit mechanical energy into tissues deep in the body without an invasive procedure. Thus, the finding that ultrasound induces the release of ATP via endogenous hemichannels in the pancreatic β-cells could pave the way for developing a new therapeutic intervention for diabetes. 

In fact, the utilization of β-cell’s endogenous purinergic signaling cascade, as an indirect pathway for ultrasound-induced Ca^2+^ signaling, gives an edge over the direct activation of Ca^2+^ influx found from other studies. First, a localized stimulation can unlock the synchronized responses from a targeted pancreas tissue via the diffusion of extracellular ATP. Second, purinergic signaling induces oscillatory, rather than sustained, Ca^2+^, which mimics β-cell’s native signaling and is also critical for the pulsatile secretion of insulin. Last, the finding that the level of cytoplasmic ATP, which essentially regulates the K_ATP_ channel-dependent GSIS pathway, also plays a pivotal role in determining the modes of oscillations in ultrasound-mediated Ca^2+^ dynamics suggests the means of intrinsic regulation.

In summary, ultrasound-mediated intracellular Ca^2+^ dynamics were investigated in HIT-T15 pancreatic β-cells using 45-MHz focused ultrasound. The results indicate that low-intensity focused ultrasound is capable of inducing distinctive oscillatory Ca^2+^ patterns from the HIT-T15 β-cell line through the endogenous purinergic signaling pathway where ATP release from mechanosensitive hemichannels works as a signaling mediator. The mechanotransduction pathway explored in this paper provides meaningful insights into the biophysical basis of ultrasound-induced Ca^2+^ dynamics in pancreatic β-cells and could stimulate further the development of a novel ultrasound-based therapeutic modality, especially against diabetic conditions. However, to claim the feasibility of the ultrasound-based Ca^2+^ modulator platform in the diabetic-related therapeutic applications, investigation on pulsatile insulin secretion upon ultrasound exposure, particularly in the context of different Ca^2+^ oscillation patterns, is necessary. The current ultrasound stimulation system is optimized for imaging live cells within an image field but lacks a populational functional assay capability. Therefore, future works will be focused on building a high-throughput stimulation system that offers consistent stimulation environment and means of systematic assessment for ultrasound-induced secretion events. Additionally, further validation with low-frequency ultrasound (2–6 MHz) is required to be more relevant to clinical settings.

## Supplementary Materials

The following are available online at https://www.mdpi.com/2073-4409/9/5/1129/s1 [58,59,60,61,62,63,64].

## References

[1] Nathan D.M., Schreiber E., Fogel H., Mojsov S., Habener J.F. (1992). Insulinotropic action of glucagonlike peptide-I-(7-37) in diabetic and nondiabetic subjects. Diabetes Care.

[2] Lundquist I., Alm P., Salehi A., Henningsson R., Grapengiesser E., Hellman B. (2003). Carbon monoxide stimulates insulin release and propagates Ca^2+^ signals between pancreatic beta-cells. Am. J. Physiol. Endocrinol. Metab..

[3] Ashcroft F.M., Rorsman P. (1990). ATP-sensitive K+ channels: A link between B-cell metabolism and insulin secretion. Biochem. Soc. Trans..

[4] Newsholme P., Gaudel C., McClenaghan N.H. (2010). Nutrient regulation of insulin secretion and beta-cell functional integrity. Adv. Exp. Med. Biol..

[5] Lang D.A., Matthews D.R., Peto J., Turner R.C. (1979). Cyclic oscillations of basal plasma glucose and insulin concentrations in human beings. N. Engl. J. Med..

[6] Bratusch-Marrain P.R., Komjati M., Waldhäusl W.K. (1986). Efficacy of pulsatile versus continuous insulin administration on hepatic glucose production and glucose utilization in type I diabetic humans. Diabetes.

[7] Matthews D.R., Lang D.A., Burnett M.A., Turner R.C. (1983). Control of pulsatile insulin secretion in man. Diabetologia.

[8] Paolisso G., Sgambato S., Torella R., Varricchio M., Scheen A., D’Onofrio F., Lefèbvre P.J. (1988). Pulsatile insulin delivery is more efficient than continuous infusion in modulating islet cell function in normal subjects and patients with type 1 diabetes. J. Clin. Endocrinol. Metab..

[9] O’Meara N.M., Sturis J., Herold K.C., Ostrega D.M., Polonsky K.S. (1995). Alterations in the patterns of insulin secretion before and after diagnosis of IDDM. Diabetes Care.

[10] Lang D.A., Matthews D.R., Burnett M., Turner R.C. (1981). Brief, irregular oscillations of basal plasma insulin and glucose concentrations in diabetic man. Diabetes.

[11] Hagren O.I., Tengholm A. (2006). Glucose and insulin synergistically activate phosphatidylinositol 3-kinase to trigger oscillations of phosphatidylinositol 3,4,5-trisphosphate in beta-cells. J. Biol. Chem..

[12] Best L., Miley H.E., Yates A.P. (1996). Activation of an anion conductance and beta-cell depolarization during hypotonically induced insulin release. Exp. Physiol..

[13] Casas S., Novials A., Reimann F., Gomis R., Gribble F.M. (2008). Calcium elevation in mouse pancreatic beta cells evoked by extracellular human islet amyloid polypeptide involves activation of the mechanosensitive ion channel TRPV4. Diabetologia.

[14] Suarez Castellanos I., Singh T., Balteanu B., Bhowmick D.C., Jeremic A., Zderic V. (2017). Calcium-dependent ultrasound stimulation of secretory events from pancreatic beta cells. J. Ther. Ultrasound.

[15] Hwang J.Y., Lee N.S., Lee C., Lam K.H., Kim H.H., Woo J., Lin M.Y., Kisler K., Choi H., Zhou Q. (2013). Investigating contactless high frequency ultrasound microbeam stimulation for determination of invasion potential of breast cancer cells. Biotechnol. Bioeng..

[16] Weitz A.C., Lee N.S., Yoon C.W., Bonyad A., Goo K.S., Kim S., Moon S., Jung H., Zhou Q., Chow R.H. (2017). Functional Assay of Cancer Cell Invasion Potential Based on Mechanotransduction of Focused Ultrasound. Front. Oncol..

[17] Hwang J.Y., Lim H.G., Yoon C.W., Lam K.H., Yoon S., Lee C., Chiu C.T., Kang B.J., Kim H.H., Shung K.K. (2014). Non-contact high-frequency ultrasound microbeam stimulation for studying mechanotransduction in human umbilical vein endothelial cells. Ultrasound Med. Biol..

[18] Yoon C.W., Jung H., Goo K., Moon S., Koo K.M., Lee N.S., Weitz A.C., Shung K.K. (2017). Low-Intensity Ultrasound Modulates Ca^2+^ Dynamics in Human Mesenchymal Stem Cells via Connexin 43 Hemichannel. Ann. Biomed. Eng..

[19] Lam K.H., Hsu H.S., Li Y., Lee C., Lin A., Zhou Q., Kim E.S., Shung K.K. (2013). Ultrahigh frequency lensless ultrasonic transducers for acoustic tweezers application. Biotechnol. Bioeng..

[20] Cannata J.M., Ritter T.A., Chen W.H., Silverman R.H., Shung K.K. (2003). Design of efficient, broadband single-element (20-80 MHz) ultrasonic transducers for medical imaging applications. IEEE Trans. Ultrason. Ferroelectr. Freq. Control..

[21] Carpenter A.E., Jones T.R., Lamprecht M.R., Clarke C., Kang I.H., Friman O., Guertin D.A., Chang J.H., Lindquist R.A., Moffat J. (2006). CellProfiler: Image analysis software for identifying and quantifying cell phenotypes. Genome Biol..

[22] Jung S.K., Kauri L.M., Qian W.J., Kennedy R.T. (2000). Correlated oscillations in glucose consumption, oxygen consumption, and intracellular free Ca(2+) in single islets of Langerhans. J. Biol. Chem..

[23] Leech C.A., Holz G.G., Habener J.F. (1994). Voltage-independent calcium channels mediate slow oscillations of cytosolic calcium that are glucose dependent in pancreatic beta-cells. Endocrinology.

[24] Lee N.S., Rohan J.G., Zitting M., Kamath S., Weitz A., Sipos A., Salvaterra P.M., Hasegawa K., Pera M., Chow R.H. (2012). A novel dual-color reporter for identifying insulin-producing beta-cells and classifying heterogeneity of insulinoma cell lines. PLoS ONE.

[25] Beauvois M.C., Merezak C., Jonas J.C., Ravier M.A., Henquin J.C., Gilon P. (2006). Glucose-induced mixed [Ca^2+^]c oscillations in mouse beta-cells are controlled by the membrane potential and the SERCA3 Ca^2+^-ATPase of the endoplasmic reticulum. Am. J. Physiol. Cell Physiol..

[26] Burnstock G. (2014). Purinergic signalling in endocrine organs. Purinergic. Signal..

[27] Tahani H.M. (1979). The purinergic nerve hypothesis and insulin secretion. Z Ernahrungswiss.

[28] Bertrand G., Chapal J., Loubatieres-Mariani M.M. (1986). Potentiating synergism between adenosine diphosphate or triphosphate and acetylcholine on insulin secretion. Am. J. Physiol..

[29] Leitner J.W., Sussman K.E., Vatter A.E., Schneider F.H. (1975). Adenine nucleotides in the secretory granule fraction of rat islets. Endocrinology.

[30] Petit P., Lajoix A.D., Gross R. (2009). P2 purinergic signalling in the pancreatic beta-cell: Control of insulin secretion and pharmacology. Eur. J. Pharm. Sci..

[31] Novak I. (2008). Purinergic receptors in the endocrine and exocrine pancreas. Purinergic. Signal..

[32] Satin L.S., Tavalin S.J., Kinard T.A., Teague J. (1995). Contribution of L- and non-L-type calcium channels to voltage-gated calcium current and glucose-dependent insulin secretion in HIT-T15 cells. Endocrinology.

[33] Bertrand G., Chapal J., Loubatieres-Mariani M.M., Roye M. (1987). Evidence for two different P2-purinoceptors on beta cell and pancreatic vascular bed. Br. J. Pharmacol..

[34] Lee D.H., Park K.S., Kim D.R., Lee J.W., Kong I.D. (2008). Dual effect of ATP on glucose-induced insulin secretion in HIT-T15 cells. Pancreas.

[35] Sabourin J., Allagnat F. (2016). Store-operated Ca^2+^ entry: A key component of the insulin secretion machinery. J. Mol. Endocrinol..

[36] Santerre R.F., Cook R.A., Crisel R.M., Sharp J.D., Schmidt R.J., Williams D.C., Wilson C.P. (1981). Insulin synthesis in a clonal cell line of simian virus 40-transformed hamster pancreatic beta cells. Proc. Natl. Acad. Sci. USA.

[37] Suarez Castellanos I., Jeremic A., Cohen J., Zderic V. (2017). Ultrasound Stimulation of Insulin Release from Pancreatic Beta Cells as a Potential Novel Treatment for Type 2 Diabetes. Ultrasound Med. Biol..

[38] Dalecki D. (2004). Mechanical bioeffects of ultrasound. Annu. Rev. Biomed. Eng..

[39] Tufail Y., Matyushov A., Baldwin N., Tauchmann M.L., Georges J., Yoshihiro A., Tillery S.I., Tyler W.J. (2010). Transcranial pulsed ultrasound stimulates intact brain circuits. Neuron.

[40] O’Reilly M.A., Huang Y., Hynynen K. (2010). The impact of standing wave effects on transcranial focused ultrasound disruption of the blood-brain barrier in a rat model. Phys. Med. Biol..

[41] Sato M., Nagayama K., Kataoka N., Sasaki M., Hane K., Kataoka N., Sasaki M., Hane K. (2000). Local mechanical properties measured by atomic force microscopy for cultured bovine endothelial cells exposed to shear stress. J. Biomech..

[42] Boitor R., Sinjab F., Strohbuecker S., Sottile V., Notingher I. (2016). Towards quantitative molecular mapping of cells by Raman microscopy: Using AFM for decoupling molecular concentration and cell topography. Faraday Discuss..

[43] Tengholm A., Gylfe E. (2009). Oscillatory control of insulin secretion. Mol. Cell Endocrinol..

[44] Grapengiesser E., Gylfe E., Hellman B. (1989). Three types of cytoplasmic Ca^2+^ oscillations in stimulated pancreatic beta-cells. Arch. Biochem. Biophys..

[45] Khakh B.S., Burnstock G., Kennedy C., King B.F., North R.A., Seguela P., Voigt M., Humphrey P.P., Seguela P., Voigt M. (2001). International union of pharmacology. XXIV. Current status of the nomenclature and properties of P_2_X receptors and their subunits. Pharmacol. Rev..

[46] Abbracchio M.P., Burnstock G., Boeynaems J.M., Barnard E.A., Boyer J.L., Kennedy C., Knight G.E., Fumagalli M., Gachet C., Jacobson K.A. (2006). International Union of Pharmacology LVIII: Update on the P_2_Y G protein-coupled nucleotide receptors: From molecular mechanisms and pathophysiology to therapy. Pharmacol. Rev..

[47] Tanaka T., Nagashima K., Inagaki N., Kioka H., Takashima S., Fukuoka H., Noji H., Kakizuka A., Imamura H. (2014). Glucose-stimulated single pancreatic islets sustain increased cytosolic ATP levels during initial Ca^2+^ influx and subsequent Ca^2+^ oscillations. J. Biol. Chem..

[48] Jacques-Silva M.C., Correa-Medina M., Cabrera O., Rodriguez-Diaz R., Makeeva N., Fachado A., Diez J., Berman D.M., Kenyon N.S., Ricordi C. (2010). ATP-gated P2 × 3 receptors constitute a positive autocrine signal for insulin release in the human pancreatic beta cell. Proc. Natl. Acad. Sci. USA.

[49] Gong Q., Kakei M., Koriyama N., Nakazaki M., Morimitsu S., Yaekura K., Tei C. (2000). P_2_Y-purinoceptor mediated inhibition of L-type Ca^2+^ channels in rat pancreatic beta-cells. Cell Struct. Funct..

[50] Qu Y., Campbell D.L., Strauss H.C. (1993). Modulation of L-type Ca^2+^ current by extracellular ATP in ferret isolated right ventricular myocytes. J. Physiol..

[51] Monaghan K.P., Koh S.D., Ro S., Yeom J., Horowitz B., Sanders K.M. (2006). Nucleotide regulation of the voltage-dependent nonselective cation conductance in murine colonic myocytes. Am. J. Physiol. Cell Physiol..

[52] McHugh D., Sharp E.M., Scheuer T., Catterall W.A. (2000). Inhibition of cardiac L-type calcium channels by protein kinase C phosphorylation of two sites in the *n*-terminal domain. Proc. Natl. Acad. Sci. USA.

[53] Bao L., Sachs F., Dahl G. (2004). Connexins are mechanosensitive. Am. J. Physiol. Cell Physiol..

[54] Garcia M., Knight M.M. (2010). Cyclic loading opens hemichannels to release ATP as part of a chondrocyte mechanotransduction pathway. J. Orthop. Res..

[55] Takada H., Furuya K., Sokabe M. (2014). Mechanosensitive ATP release from hemichannels and Ca^2+^ influx through TRPC6 accelerate wound closure in keratinocytes. J. Cell Sci..

[56] Batra N., Burra S., Siller-Jackson A.J., Gu S., Xia X., Weber G.F., DeSimone D., Bonewald L.F., Lafer E.M., Sprague E. (2012). Mechanical stress-activated integrin alpha5beta1 induces opening of connexin 43 hemichannels. Proc. Natl. Acad. Sci. USA.

[57] Lee N.S., Yoon C.W., Wang Q., Moon S., Koo K.M., Jung H., Chen R., Jiang L., Lu G., Fernandez A. (2020). Focused ultrasound stimulates ER localized mechanosensitive PANNEXIN-1 to mediate intracellular calcium release in invasive cancer cells. BioRxiv.

[58] Orriss I.R., Key M.L., Hajjawi M.O., Arnett T.R. (2013). Extracellular ATP released by osteoblasts is a key local inhibitor of bone mineralisation. PLoS ONE.

[59] Hoyle C.H., Knight G.E., Burnstock G. (1990). Suramin antagonizes responses to P2-purinoceptor agonists and purinergic nerve stimulation in the guinea-pig urinary bladder and taenia coli. Br J Pharmacol.

[60] Anselmi F., Hernandez V.H., Crispino G., Seydel A., Ortolano S., Roper S.D., Kessaris N., Richardson W., Rickheit G., Filippov M.A. (2008). ATP release through connexin hemichannels and gap junction transfer of second messengers propagate Ca^2+^ signals across the inner ear. Proc. Natl. Acad. Sci. USA.

[61] Flores-Soto E., Reyes-Garcia J., Sommer B., Chavez J., Barajas-Lopez C., Montano L.M. (2012). PPADS, a P_2_X receptor antagonist, as a novel inhibitor of the reverse mode of the Na^+^/Ca^2+^ exchanger in guinea pig airway smooth muscle. Eur. J. Pharmacol..

[62] Seidler N.W., Jona I., Vegh M., Martonosi A. (1989). Cyclopiazonic acid is a specific inhibitor of the Ca^2+^-ATPase of sarcoplasmic reticulum. J. Biol. Chem..

[63] Nobile M., Monaldi I., Alloisio S., Cugnoli C., Ferroni S. (2003). ATP-induced, sustained calcium signalling in cultured rat cortical astrocytes: Evidence for a non-capacitative, P_2_X7-like-mediated calcium entry. FEBS Lett..

[64] Tian C., Du L., Zhou Y., Li M. (2016). Store-operated CRAC channel inhibitors: Opportunities and challenges. Future Med. Chem..

