# Supplementary material for: Investigation of Ultrasound-Mediated Intracellular Ca2+ Oscillations in HIT-T15 Pancreatic β-Cell Line
Source: Cells. 2020 May 4;9(5):1129. doi: 10.3390/cells9051129 (PMC7290496; doi:10.3390/cells9051129)
Supplement: Supplementary file 1 [file cells-09-01129-s001.zip › Supplementary_Yoon/Supplementary.docx]

Supplementary Information

**Supplementary Video 1:** **Ultrasound-induced intracellular Ca^2+^ oscillations from HIT-T15 pancreatic β-cells.** Clusters of HIT-T15 cells were exposed to low-intensity pulsed ultrasound for 25 min starting at t = 300 s. Fast-irregular oscillations were dominantly observed as cells were bathed in HBSS+ (11.1. mM glucose).

**Supplementary Table 1:** **Pharmaceutical agents utilized in this study.**

| Agent | Effect | Conc. (μM) | Loading Time (min) | Reference |
| --- | --- | --- | --- | --- |
| Apyrase | Depletion of extracellular ATP | 20 units/mL | 10 | [1] |
| Suramin | A non-selective P_2_ receptor antagonist | 100 | 10 | [2, 3] |
| CBX | A gap junction/hemichannel blocker | 100 | 30 | [4] |
| PPADS | A selective P_2_X receptor inhibitor | 100 | 30 | [3, 5] |
| Nifedipine | A L-type Ca^2+^ channel blocker | 10 | 30 | [6, 7] |
| CPA | Depletion of ER Ca^2+^ storage | 100 | 30 | [8, 9] |
| La^3+^ | A CRAC channel blocker | 200 | 0 | [7, 10] |


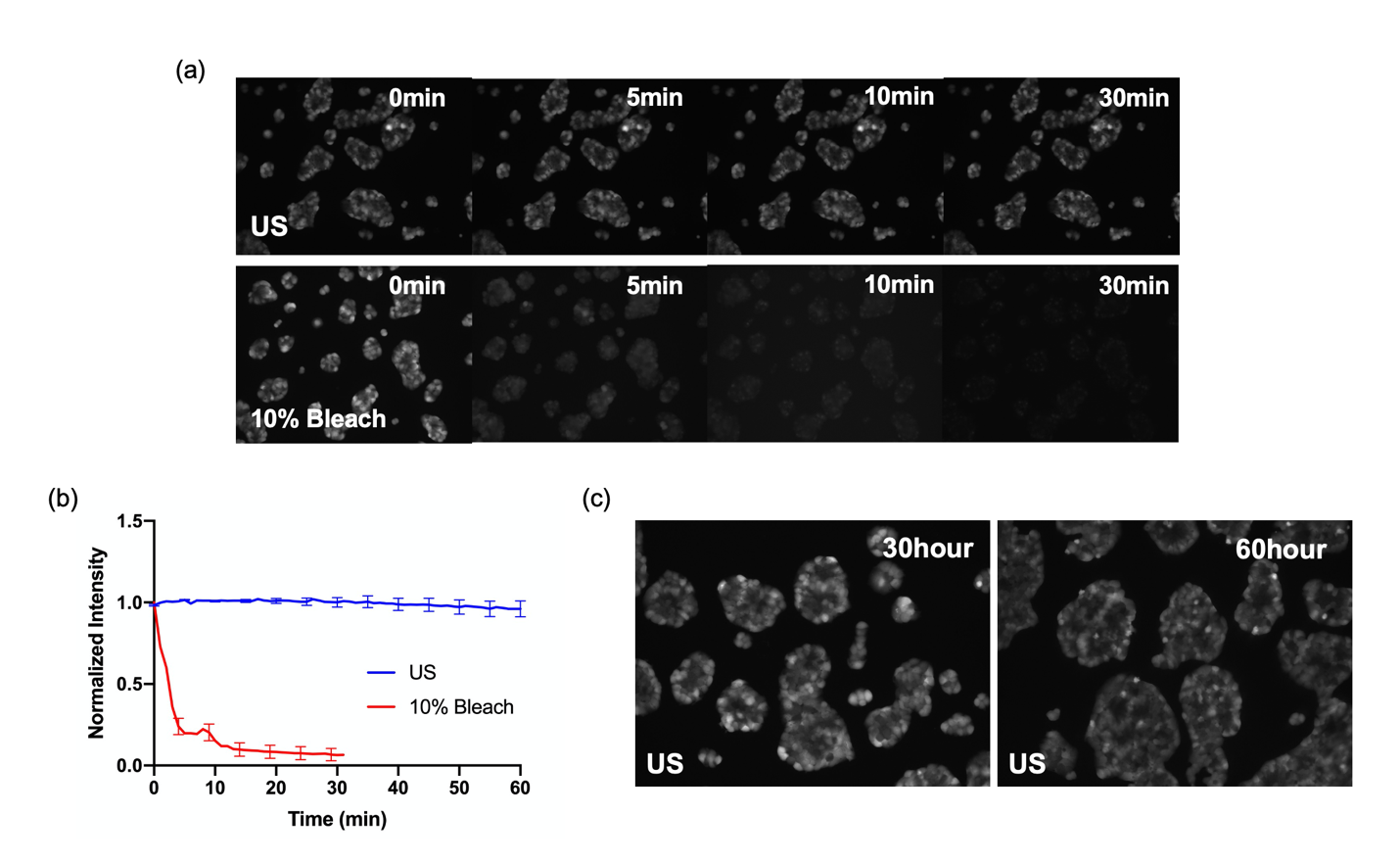


**Supplementary Figure 1.** **Viability assay upon ultrasound stimulation.** Cytotoxicity of ultrasound was measured using a widely used viability dye, Calcien AM (Thermofisher). The cells bathed with 10% bleach quickly lost its membrane integrity resulted in decrease of fluorescence intensity. In contrast, the cells exposed to ultrasound (30 min, I_SPTA_: 113.1 mW/cm^2^) was not affected. The cells exposed to ultrasound was also assayed 30 and 60 hours after the stimulation to check long term effects. Still no sign of cytotoxicity was observed.


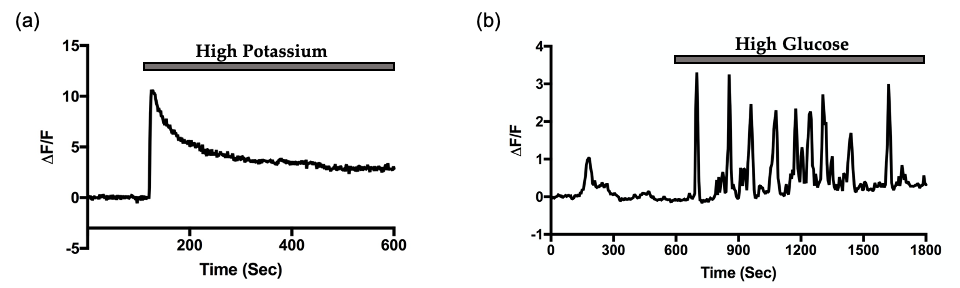


**Supplementary Figure 2.** **Intracellular Ca^2+^ dynamics were triggered by secretagogues**. (a) High potassium (40 mM) buffer or (b) high glucose (17 mM) buffer were added while imaging the Ca^2+^ dynamics from the HIT-T15 cells. The high potassium stimulation induces abrupt increase of intracellular Ca^2+^ followed by gradual decrease, and the high glucose stimulation induces oscillatory Ca^2+^ response.

# References

1. Orriss, I.R., et al., *Extracellular ATP released by osteoblasts is a key local inhibitor of bone mineralisation.* PLoS One, 2013. **8**(7): p. e69057.

2. Hoyle, C.H., G.E. Knight, and G. Burnstock, *Suramin antagonizes responses to P2-purinoceptor agonists and purinergic nerve stimulation in the guinea-pig urinary bladder and taenia coli.* Br J Pharmacol, 1990. **99**(3): p. 617-21.

3. Gong, Q., et al., *P2Y-purinoceptor mediated inhibition of L-type Ca2+ channels in rat pancreatic beta-cells.* Cell Struct Funct, 2000. **25**(5): p. 279-89.

4. Anselmi, F., et al., *ATP release through connexin hemichannels and gap junction transfer of second messengers propagate Ca2+ signals across the inner ear.* Proc Natl Acad Sci U S A, 2008. **105**(48): p. 18770-5.

5. Flores-Soto, E., et al., *PPADS, a P2X receptor antagonist, as a novel inhibitor of the reverse mode of the Na(+)/Ca(2)(+) exchanger in guinea pig airway smooth muscle.* Eur J Pharmacol, 2012. **674**(2-3): p. 439-44.

6. Satin, L.S., et al., *Contribution of L- and non-L-type calcium channels to voltage-gated calcium current and glucose-dependent insulin secretion in HIT-T15 cells.* Endocrinology, 1995. **136**(10): p. 4589-601.

7. Leech, C.A., G.G.t. Holz, and J.F. Habener, *Voltage-independent calcium channels mediate slow oscillations of cytosolic calcium that are glucose dependent in pancreatic beta-cells.* Endocrinology, 1994. **135**(1): p. 365-72.

8. Seidler, N.W., et al., *Cyclopiazonic acid is a specific inhibitor of the Ca2+-ATPase of sarcoplasmic reticulum.* J Biol Chem, 1989. **264**(30): p. 17816-23.

9. Nobile, M., et al., *ATP-induced, sustained calcium signalling in cultured rat cortical astrocytes: evidence for a non-capacitative, P2X7-like-mediated calcium entry.* FEBS Lett, 2003. **538**(1-3): p. 71-6.

10. Tian, C., et al., *Store-operated CRAC channel inhibitors: opportunities and challenges.* Future Med Chem, 2016. **8**(7): p. 817-32.
